# Loss of CAR promotes migration and proliferation of HaCaT cells, and accelerates wound healing in rats via Src-p38 MAPK pathway

**DOI:** 10.1038/srep19735

**Published:** 2016-01-25

**Authors:** Linlin Su, Lanqing Fu, Xiaodong Li, Yue Zhang, Zhenzhen Li, Xue Wu, Yan Li, Xiaozhi Bai, Dahai Hu

**Affiliations:** 1 Department of Burns and Cutaneous Surgery, Xijing Hospital, the Fourth Military Medical University, Xi’an, Shaanxi 710032, China; 2 Department of Orthopedics, Jingzhou Central Hospital, Tongji Medical College of Huazhong University of Science and Technology, Jingzhou, Hubei 434020, China; 3 Department of Burns and Plastic Surgery, General Hospital of Lanzhou Petrochemical Company, Lanzhou, Gansu 730060, China

## Abstract

The coxsackie and adenovirus receptor (CAR) is a cell adhesion molecule mostly localized to cell-cell contacts in epithelial and endothelial cells. CAR is known to regulate tumor progression, however, its physiological role in keratinocyte migration and proliferation, two essential steps in re-epithelialization during wound healing, has less been investigated. Here we showed that CAR was predominantly expressed in the epidermis of human skin, CAR knockdown by RNAi significantly accelerated HaCaT cell migration and proliferation. In addition, knockdown of CAR *in vitro* increased *p*-Src, *p*-p38, and *p*-JNK protein levels; however, Src inhibitor PP2 prevented the increase of *p*-Src and *p*-p38 induced by CAR RNAi, but not *p*-JNK, and decelerated cell migration and proliferation. More intriguingly, *in vivo* CAR RNAi on the skin area surrounding the wounds on rat back visually accelerated wound healing and re-epithelialization process, while treatment with PP2 or p38 inhibitor SB203580 obviously inhibited these effects. By contrast, overexpressing CAR in HaCaT cells significantly decelerated cell migration and proliferation. Above results demonstrate that suppression of CAR could accelerate HaCaT cell migration and proliferation, and promote wound healing in rat skin, probably via Src-p38 MAPK pathway. CAR thus might serve as a novel therapeutic target for facilitating wound healing.

Skin wound healing is a multifaceted process of re-epithelialization that requires epidermal cell migration and proliferation, collagen fiber rearrangement, and cutaneous adnexa repair^1^. CAR, a 46-kD transmembrane protein, has been implicated in the regulation of cancer metastasis and development, and was found to exist in mouse skin keratinocytes^2^. However, its involvement in wound healing has less been investigated, let alone the underlying mechanism.

CAR was first characterized in epithelial cells^3^ and was later identified as an integral component of tight junction^4^. In several human carcinomas, CAR has been shown to regulate cancer cell adhesion, proliferation, migration and invasion. Whereas their normal tissue counterparts express readily detectable levels of CAR, many tumor tissues or cell lines only have little CAR expression^5^. Loss of CAR has been implicated to promote the proliferation, migration and invasion of cancer cells^6^, while the enhanced expression of CAR reduces tumor migration and metastasis in human prostate cancer^7^, bladder cancer^8^ and glioma cell lines^9^. Additionally, CAR has been shown to mediate the trans-endothelial migration of neutrophils^10^ and the passage of migratory germ cell cross the blood-testis barrier^11^. Therefore in this study, we hypothesize that CAR regulates epidermal cell migration, proliferation and wound healing, and further explore the involved signaling.

Src belongs to Src family kinases which include nine non-receptor protein tyrosine kinases expressed ubiquitously and are essential for numerous cellular processes such as proliferation, migration and transformation. Src is activated via three ways: phosphorylation at Tyr416 residue, dephosphorylation at Tyr527 residue, or combination with certain receptors (e.g. growth factor receptor)^12^. Src has been implicated in regulating signaling pathways related to cell migration and proliferation, such as Akt, STAT3 phosphorylation^13^ and Ras activation^14^. Besides, there are growing evidences showing Src involvement in activating MAPK^15^. Three major groups of MAPK cascades: Erk1/2, JNK and p38 MAPK, with activation sites at Thr202/Tyr204, Thr183/Tyr185 and Thr180/Tyr182, respectively, are implicated in the regulation of multiple cellular behaviors, such as cell migration and proliferation^16^.

Therefore, we hypothesize that CAR could regulate epidermal cell migration, proliferation, and wound healing, at least in part, through Src-MAPK pathway. To test this hypothesis, we utilized HaCaT cells, an immortalized human keratinocyte line, and wounded rats on the back skin as *in vitro* and *in vivo* models in this study, respectively. We then exploited RNAi technique alone or combination with drug treatment, such as PP2, a putative Src inhibitor^17^, and SB203580, a p38 inhibitor, to investigate the mechanisms underlying CAR’s regulation on cell migration, proliferation, and *in vivo* wound healing. Finally, we included CAR overexpression to confirm above findings from another perspective. Our results showed that repression of CAR expression could stimulate keratinocyte migration, proliferation, and *in vivo* wound healing probably via Src-p38 MAPK pathway, thus CAR might serve as a potential molecular target to promote wound healing.

## Results

### CAR is predominantly expressed in the epidermis of the skin

**Figure 1:**
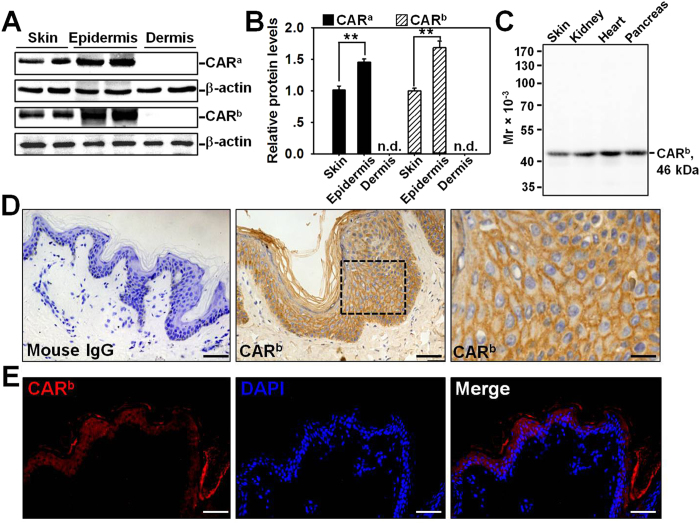
Expression and localization of CAR in normal human skin. (**A**) Immunoblotting of CAR in lysates from normal human skin, epidermis, and dermis (~40 μg proteins/lane) with β-actin serving as a loading control. Two antibodies were utilized, anti-CAR^a^ is a rabbit origin antibody, while anti-CAR^b^ is a mouse origin antibody that was used in all following experiments. (**B**) The CAR level in skin after normalization against its corresponding β-actin was arbitrarily set at 1. Error bars represent means ± SD from four individuals (*n* = 4); **p < 0.01; *n.d.*, not detectable. (**C**) Specificity of the mouse anti-CAR^b^ antibody was demonstrated by an immunoblot using normal human skin, kidney, heart, and pancreas lysates. (**D**) Immunohistochemistry staining of CAR on normal human skin paraffin section, in which the positive CAR staining appeared as brownish precipitates (*middle and right images*). Image on the *right* was the enlargement of the boxed area in the *middle* image. Sections incubated with normal mouse IgG instead of the mouse anti-CAR served as negative control (*left*). Scale bars: *left*, 50 μm; *middle,* 30 μm; *right*, 10 μm. (**E**) Immunohistofluorescent staining of CAR (*red*) on normal human skin paraffin section. DAPI was used for nuclear staining. Scale bar: 50 μm.

CAR is known to regulate tumor progression and metastasis, thus we are interested to investigate if CAR is also involved in skin wound healing. We first examined the expression pattern of CAR in normal human skin, epidermis, and dermis by western blot using two different anti-CAR antibodies, one is rabbit origin and designated as anti-CAR^a^, the other is mouse origin and designated as anti-CAR^b^ (Table S1). The two antibodies revealed the same CAR expression pattern: CAR protein level in the epidermis was 1.5~1.7-fold higher than that in the skin, while not detectable in the dermis (Fig. 1A,B). Samples from normal human skin, kidney, heart, and pancreas were included to evaluate the specificity of anti-CAR^b^ by western blot. All four tissues expressed moderate level of CAR, and anti-CAR^b^ is suitable for following staining experiments due to its specificity (Fig. 1C). Immunohistochemistry (IHC) on normal skin paraffin section using anti-CAR^b^ clearly showed that CAR was predominantly distributed in the epidermis, concentrating at the cell-cell contacts which is in accordance with the finding that CAR is a putative tight junction protein, while absent in the dermis (Fig. 1D). Immunohistofluorescence (IHF) staining further confirmed CAR’s localization in the epidermis, but not the dermis of the skin (Fig. 1E). IHC and IHF experiments with the use of rabbit anti-CAR^a^ antibody did not work, thus we used the anti-CAR^b^ for all following studies. Above results demonstrate that CAR is predominantly distributed in the epidermis of normal human skin.

### CAR knockdown by RNAi accelerates HaCaT cell proliferation and migration

We know that the proper proliferation and timely migration of epidermal cells (keratinocytes) are essential for normal wound healing, thus next we went to seek a suitable *in vitro* cell model to study the regulation of CAR on cell proliferation and migration. However, the use of primary keratinocytes has been hindered by the stringent culture requirements and limitations imposed by the inherent properties of the cells due to their short lifespan, while transformed cell lines usually exhibit phenotypic features not found in normal cells. In contrast, the spontaneously immortalized HaCaT cell line has been a widely employed keratinocyte model due to its ease of propagation and near normal phenotype, and thus selected in our study.

**Figure 2:**
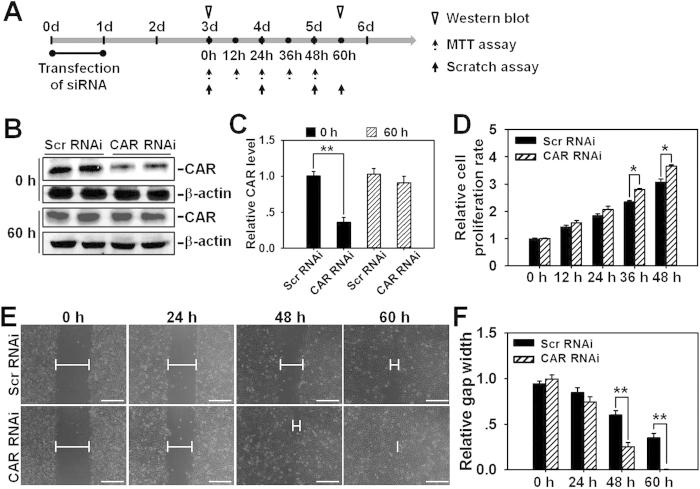
Study by RNAi to assess the effect of CAR knockdown on HaCaT cell migration and proliferation. (**A**) Regimen used in RNAi experiment *in vitro*. Briefly, HaCaT cells were transfected with either CAR-specific or scramble siRNA duplexes for 24 h. Two days after the completion of RNAi (namely on day 3), western blot, scratch assay, and MTT assay were conducted at selective time points. (**B**) Representative immunoblots illustrating CAR protein levels at 0 h (day 3) and 60 h (day 5.5) post-RNAi treatment. (**C**) CAR level in Scramble (Scr) RNAi group after normalization against corresponding β-actin was arbitrarily set at 1. Error bars represent means ± SD from four independent experiments using different batches of cells (*n* = 4), **p < 0.01. (**D**) MTT assay was performed on day 3 following RNAi to assess the effects of CAR knockdown on HaCaT cell proliferation at selective time points of 0, 12, 24, 36, and 48 h. Error bars represent means ± SD from four different batches of cells (*n* = 4), *p < 0.05. (**E**) Scratch assay illustrating the effects of CAR knockdown on cell migration. On day 3 post-RNAi treatment, HaCaT cells received 1-h mitomycin C treatment, and then subjected to scratch assay. Images were taken at 0, 24, 48, and 60 h post-scratching. Blunted lines indicate the average width of unclosed gap. Scale bar: 100 μm. (**F**) Scratch gap width at 0 h in each group was arbitrarily set at 1. Error bars represent means ± SD from four independent experiments using different batches of cells (*n* = 4), **p < 0.01.

Next we conducted RNAi in HaCaT cells to specifically suppress CAR expression and further observe the effect of CAR knockdown on cell proliferation and migration. Briefly, HaCaT cells were subjected to a 24-h RNAi transfection on day 0, then CAR expression level, cell proliferation, and migration were examined on day 3 by western blot, MTT assay, and scratch assay, respectively, several time points for each experiment were selected as shown in Fig. 2A. Western blot analysis showed that CAR protein level decreased by ~70% after CAR knockdown on day 3 at 0 h (i.e., two days after the completion of RNAi transfection), while restored almost to the normal level at 60 h (on day 5.5) (Fig. 2B,C). MTT assay revealed the accelerated cell proliferation rate in CAR RNAi-transfected HaCaT cells (Fig. 2D). In scratch assay, HaCaT cells were pretreated with 1-h mitomycin C to inhibit cell proliferation so that we could observe net effect of CAR knockdown on cell migration. Results showed that loss of CAR by *in vitro* RNAi significantly accelerated cell migration and shortened the time course needed for gap closure (Fig. 2E,F). At 60 h post-scratching, the scratch gap in CAR RNAi-treated cells were fully closed (Fig. 2E,F). Above results demonstrate that the suppression of CAR expression remarkably promotes HaCaT cell proliferation and migration.

### CAR knockdown induces the phosphorylation of Src, JNK, and p38 in HaCaT cells

**Figure 3:**
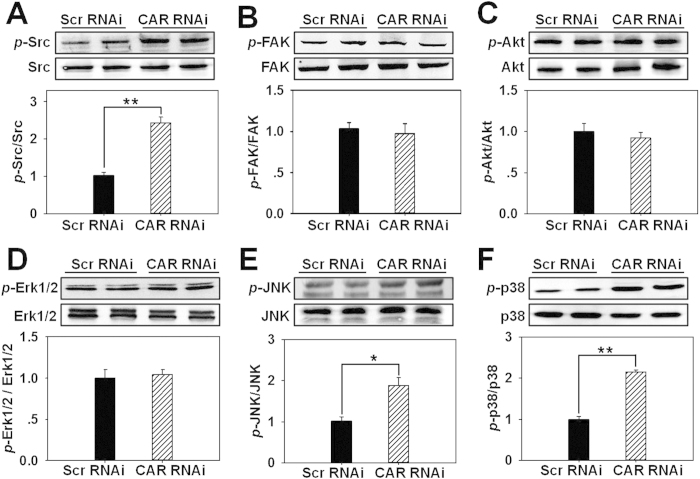
Assessment of the expression of several signaling molecules and their corresponding phosphorylated forms following CAR knockdown in HaCaT cells. Immunoblots showing the protein level changes of *p*-Src and Src (**A**), *p*-FAK and FAK (**B**), *p*-Akt and Akt (**C**), *p*-Erk1/2 and Erk1/2 (**D**), *p*-JNK and JNK (**E**), as well as *p*-p38 and p38 (**F**) in HaCaT cells after RNAi treatment. Densitometric value for each phosphorylation was normalized against its corresponding total protein and shown as mean ± SD from four different batches of cells (*n* = 4), *p < 0.05, **p < 0.01. ‘Scr’, scramble.

To explore the underlying mechanism on CAR knockdown-induced acceleration of cell proliferation and migration, we examined the expression of several related signaling molecules and their corresponding phosphorylated forms following CAR knockdown in HaCaT cells. Western blot analysis showed that the total protein level of each examined molecule did not change, while the phosphorylation of Src (Fig. 3A), JNK (Fig. 3E), and p38 (Fig. 3F) significantly increased by 2.3, 1.8, and 2.2 folds, respectively, the phosphorylated levels of FAK (Fig. 3B), Akt (Fig. 3C), and Erk1/2 (Fig. 3D) did not change, suggesting the activation of Src, JNK, and p38 following CAR knockdown. These results were further confirmed by immunocytofluorescence showing the enhanced staining of *p*-Src (Fig. S1B), *p*-JNK (Fig. S1C), and *p*-p38 (Fig. S1D) in CAR RNAi-transfected HaCaT cells. After RNAi, CAR almost vanished at HaCaT cell-cell interface demonstrating the efficient CAR knockdown *in vitro* (Fig. S1A). Moreover, in scramble RNAi-transfected cells, *p*-Src (Fig. S1B), *p*-JNK (Fig. S1C), and *p*-p38 (Fig. S1D) slightly located at cell cytosol or nuclei, while in CAR RNAi-transfected cells, they dramatically surged in cell cytosol, nuclei, and/or membrane (Fig. S1B-D). Above results demonstrate that Src, JNK, and p38 might be involved in CAR knockdown-induced acceleration of HaCaT cell proliferation and migration.

### The inhibition of Src by PP2 differentially influences p38 and JNK signaling

**Figure 4:**
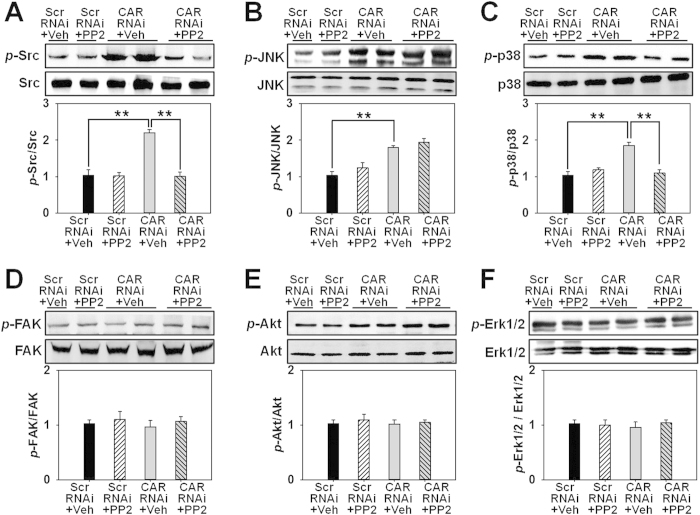
Effect of PP2 pretreatment on CAR knockdown-induced expression change of several signaling proteins and their corresponding phosphorylated forms in HaCaT cells. HaCaT cells were pretreated with vehicle (DMSO) or PP2 (an Src inhibitor) for 2 h before subjected to RNAi treatment. Cell lysates were harvested and analyzed for immunoblot analysis of *p*-Src (**A**), *p*-JNK (**B**), *p*-p38 (**C**), *p*-FAK (**D**), *p*-Akt (**E**), and *p*-Erk1/2 (**F**) as well as their corresponding total proteins. Densitometric value for each phosphorylation was normalized against its corresponding total protein and shown as mean ± SD from four different batches of cells (*n* = 4), **p < 0.01. ‘Scr’, scramble; ‘Veh’, vehicle.

Next we utilized PP2, a putative inhibitor for Src, to investigate any change on other signaling molecules. Western blot analysis revealed that PP2 significantly prevented *p*-Src up-regulation induced by CAR RNAi (Fig. 4A), showing its effectiveness as an Src inhibitor in this study. Then we examined whether Src inhibition by PP2 would affect MAPK signaling. Results exhibited that PP2 notably inhibited the up-regulated *p*-p38 level induced by CAR RNAi (Fig. 4C), while did not affect *p*-JNK level (Fig. 4B). The protein level of *p*-FAK (Fig. 4D), *p*-Akt (Fig. 4E), or *p*-Erk1/2 (Fig. 4F) showed no response no matter to CAR RNAi alone or with PP2 treatment. Immunocytofluorescence further confirmed above results, showing weakened immunostaining of *p*-Src (Fig. S2A) and *p*-p38 (Fig. S2C) by PP2 treatment in CAR-RNAi transfected HaCaT, while *p*-JNK staining showed no difference after PP2 treatment (Fig. S2B). Above results demonstrate that p38 but not JNK is a potential downstream molecule of Src in CAR knockdown-mediated signaling.

### The inhibition of Src by PP2 or inhibition of p38 by SB203580 abolishes the accelerated HaCaT cell migration and proliferation induced by CAR knockdown

**Figure 5:**
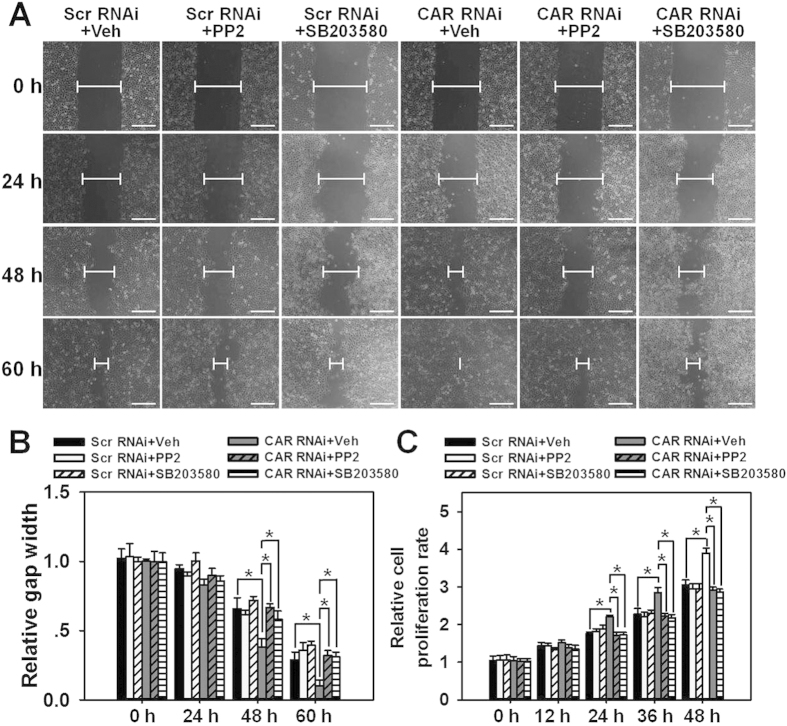
Study to assess the effect of PP2 or SB203580 pretreatment on CAR knockdown-induced acceleration of cell migration and proliferation in HaCaT cells. (**A**) HaCaT cells were pretreated with vehicle (DMSO), PP2 or SB203580 (a p38 inhibitor) for 2 h before RNAi. Before scratch wound was made, cells received 1-h mitomycin C treatment to inhibit cell proliferation. The width of each scratch gap was monitored at 0, 24, 48, and 60 h post-scratching. Blunted lines indicate the average width of unclosed gap. Scale bar:100 μm. (**B**) Scratch gap at 0 h in each treatment group was arbitrarily set at 1. Error bars represent means ± SD from four independent experiments using different batches of cells (*n* = 4), *p < 0.05. (**C**) MTT assay was conducted after cells receiving drug and RNAi treatments at designated time points of 0, 12, 24, 36, and 48h. Error bars represent means ± SD from four independent experiments using different batches of cells (*n* = 4), *p < 0.05. ‘Scr’, scramble; ‘Veh’, vehicle.

To further demonstrate the involvement of Src and p38 in CAR knockdown-induced acceleration of cell migration and proliferation, scratch assay and MTT assay were performed with the use of Src inhibitor PP2 and p38 inhibitor SB203580. Results showed that both PP2 and SB203580 led to significant retardation of scratch gap closure (Fig. 5A,B) and attenuation of cell proliferation (Fig. 5C) in CAR RNAi-transfected HaCaT cells. Above results further confirm the participation of Src and p38 in CAR-mediated HaCaT cell migration and proliferation.

### *In vivo* knockdown of CAR accelerates wound healing in adult rat

**Figure 6:**
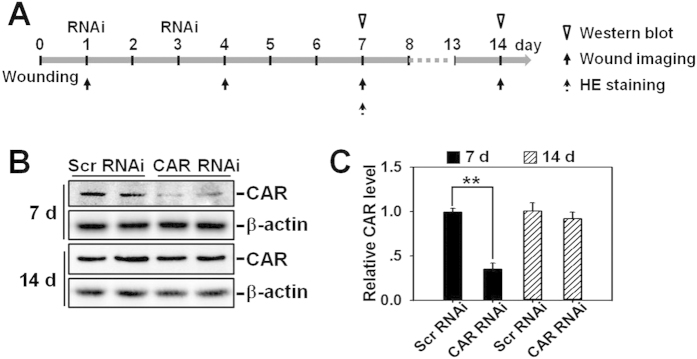
Effect of *in vivo* RNAi on CAR protein level in adult rat skin. (**A**) Regimen used in RNAi experiment *in vivo*. Briefly, 1.0 cm^2^ full-thickness excision wound was made on the mid-back of rats on day 0, skin tissue surrounding the wound was then locally injected with scramble or CAR-specific siRNA duplex on day 1 and 3. (**B**) Skin tissues surrounding wounds from day-7 and day-14 rats were excised and subjected to immunoblotting. β-Actin served as an equal loading control. (**C**) The CAR level in each Scratch RNAi group was set at 1. Error bars represent means ± SD from four different rats (*n* = 4), **p < 0.01. ‘Scr’, scramble.

**Figure 7:**
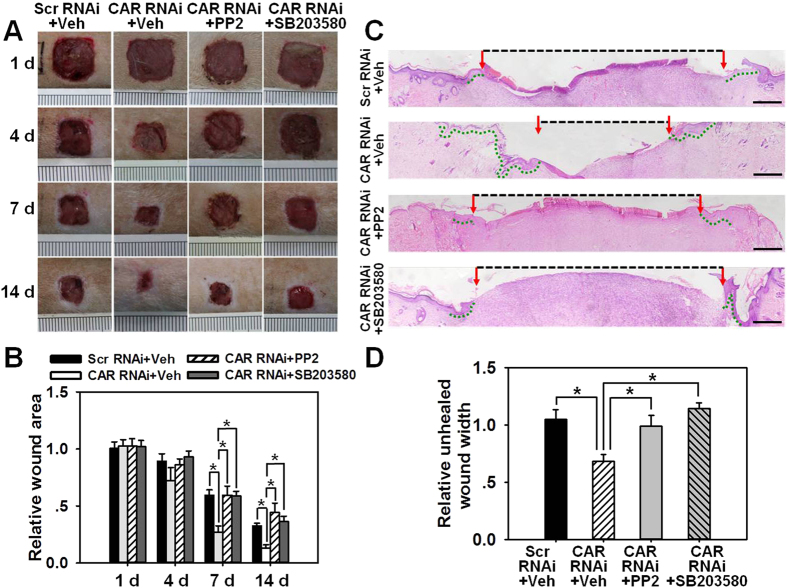
Study to assess the effect of CAR knockdown on wound healing in rats. 1.0 cm^2^ full-thickness excision wounds were made on the mid-back of rats on day 0, skin tissue surrounding the wound then topically received RNAi transfection along with vehicle (Veh, DMSO), PP2 or SB203580 on day 1 and 3. (**A**) Representative day-1, 4, 7 and 14 wounds.The wound area in CAR RNAi + Veh group was significantly reduced from day-4 post-wounding, while this effect was abolished by PP2 or SB203580. Ruler notches = 1 mm. (**B**) Wound area on day-1 in each treatment group was arbitrarily set at 1. Error bars represent means ± SD from four independent experiments using different rats (*n* = 4), *p < 0.05. (**C**) HE-stained sections of day-7 wounds were photographed to compare re-epithelialization and unhealed wound gap among each group. Multiple overlapping pictures were used to reconstitute the entire necessary part of skin wounds. Red arrows point to the leading edge of newly formed epidermis. Green-dotted lines indicate the re-epithelialized epidermis. Black-dash lines indicate unhealed wound gap. Scale bar: 400 μm. (**D**) showing the relative unhealed wound width among each group. Error bars represent means ± SD from four different rats (*n* = 4), *p < 0.05. ‘Scr’, scramble; ‘Veh’, vehicle.

The ultimate question is whether CAR knockdown is able to expedite wound healing *in vivo* and whether this is mediated *via* Src-p38 signaling as demonstrated by above *in vitro* experiments. Rats were wounded on the mid-back on day 0, then received RNAi treatment in the epidermis of skin surrounding the wounds on day 1 and day 3, followed by western blot, wound imaging, or H&E staining at designated time points as shown in Fig. 6A. On day 7 and day 14 post-wounding, skins receiving RNAi treatment were collected and subjected to western blot analysis, which showed an ~60% off on CAR protein level in CAR RNAi-treated rats skin on day 7, however, CAR level rebounded on day 14 and showed no difference with scramble RNAi-treated rat skin (Fig. 6B,C). To observe the effect of CAR knockdown on wound healing more visually, wounds in each treatment group were continuously photographed, recorded and analyzed on day 1, 4, 7, and 14 post-wounding. It is worthwhile to note that PP2 or SB203580, the Src or p38 inhibitor, respectively, was injected along with siRNA duplexes on day 1 and day 3. Results showed that CAR RNAi alone significantly promoted wound closure from day 7, while the co-administration of CAR RNAi with PP2 or SB203580 remarkably slowed the healing process (Fig. 7A,B). H&E staining using day-7 wounds revealed more re-epithelialization (indicated by green-dotted lines) and shorter wound gap (indicated by black-dash lines between two red arrows) in CAR RNAi-treated wounds, while the co-injection of CAR RNAi with PP2 or SB203580 completely abolished the accelerated re-epithelialization process (Fig. 7C,D). Above results demonstrate that CAR knockdown *in vivo* could promote rat skin wound healing probably *via* Src-p38 signaling.

### Overexpression of CAR in HaCaT cells attenuates cell proliferation and migration

**Figure 8:**
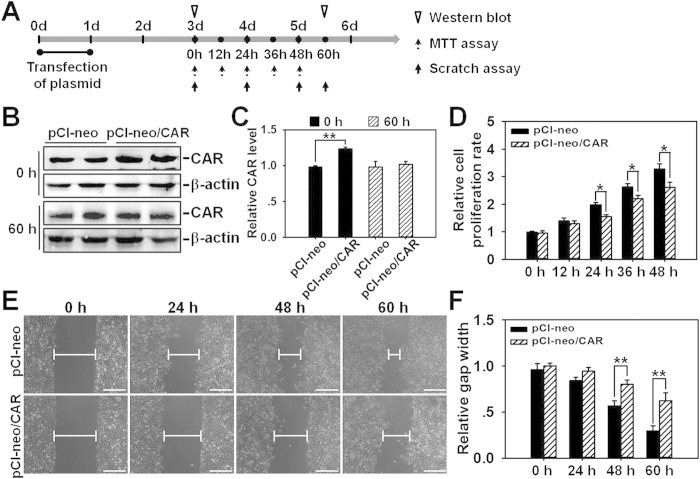
Study to assess the effect of CAR overexpression on HaCaT cell migration and proliferation. (**A**) Regimen used in gene overexpression experiment *in vitro*. Briefly, HaCaT cells were transfected with either pCI-neo alone or pCI-neo/CAR plasmid for 24 h. Two days after the completion of transfection (namely on day 3), western blot, scratch assay, and MTT assay were conducted at selective time points. (**B**) Representative immunoblots illustrating CAR protein levels at 0 h (day 3) and 60 h (day 5.5) after the transfection of overexpression vector. (**C**) CAR level in pCI-neo alone group after normalization against corresponding β-actin was arbitrarily set at 1. Error bars represent means ± SD from four independent experiments using different batches of cells (*n* = 4), **p < 0.01. (**D**) MTT assay was performed on day 3 to assess the effect of CAR overexpession on HaCaT cell proliferation at selective time points of 0, 12, 24, 36, and 48 h. Error bars represent means ± SD from four different batches of cells (*n* = 4), *p < 0.05. (**E**) Scratch assay illustrating the effect of CAR overexpession on HaCaT cell migration. On day 3 post-plasmid transfection, cells first received 1-h mitomycin C treatment, and then subjected to scratch assay. Images were taken at 0, 24, 48, and 60 h post-scratching. Blunted lines indicate the average width of unclosed gap. Scale bar: 100 μm. (**F**) The scratch gap width at 0 h in each group was arbitrarily set at 1. Error bars represent means ± SD from four independent experiments using different batches of cells (*n* = 4), **p < 0.01.

Finally, we performed CAR overexpression in HaCaT cells to confirm above results obtained from RNAi. Briefly, HaCaT cells were subjected to a 24-h transfection of pCI-neo empty vector or pCI-neo/CAR on day 0, then the CAR expression level, cell proliferation, and migration were examined on day 3 by western blot, MTT assay, and scratch assay, respectively, several time points for each experiment were selected as shown in Fig. 8A. Western blot analysis showed that CAR protein level increased by ~25% in pCI-neo/CAR-transfected HaCaT cells on day 3 at 0 h (i.e., two days after the completion of plasmid transfection), while restored to the same level as in pCI-neo-transfected HaCaT cells at 60 h (on day 5.5) (Fig. 8B,C). MTT assay revealed the decelerated cell proliferation rate in pCI-neo/CAR-transfected HaCaT cells (Fig. 8D). In scratch assay, HaCaT cells were pretreated with 1-h mitomycin C to inhibit cell proliferation so that we could observe net effect of CAR overexpression on cell migration. Results showed that overexpression of CAR significantly decelerated cell migration and prolonged the time course needed for gap closure (Fig. 2E,F). Above results demonstrate that the up-regulation of CAR expression remarkably slows down HaCaT cell proliferation and migration.

## Discussion

Multiple epithelial and endothelial cells in mammalian body are known to express CAR as a structural protein and/or cell adhesion molecule at intercellular junctions, such as tight junction^18^. Emerging evidences have shown that CAR’s function in cellular physiology is far beyond that mentioned above. For example, with increasing malignancy marked as uncontrolled metastasis and proliferation, tumors progressively lose CAR expression as compared with adjacent normal cells^19^. By contrast, CAR overexpression was shown to decrease tumor cell proliferation^9^, CAR was also found to reduce the metastatic potential of murine lung cancer^20^. Above findings demonstrate the involvement of CAR in oncogenesis.

In this study, CAR was for the first time shown to negatively regulate HaCaT cell (an immortalized human keratinocyte line) migration, proliferation, and *in vivo* wound healing, the underlying mechanism was at least partially clarified. We have demonstrated that CAR predominantly exists in the epidermis of normal human skin at the cell-cell contacts (Fig. 1), confirming its role as a junctional molecule. Such a distribution pattern is important for CAR as it has laid a foundation for further function and mechanism study. Knockdown of CAR in HaCaT cells *in vitro* was shown to accelerate cell migration and proliferation (Fig. 2), accompanied by a significant activation of Src, p38, and JNK (Fig. 3 and Fig. S1), suggesting the possible involvement of Src, JNK, and p38 MAPK signaling in CAR RNAi-mediated acceleration of cell migration and proliferation. Putative inhibition of Src by PP2 abolished the increase of p38 phosphorylation but not *p*-JNK (Fig. 4 and S2), suggesting that Src was not likely to be critical for JNK activation. Src inhibition by PP2 and p38 inhibition by SB203580 also resulted in retarded cell migration (Fig. 5A,B) and proliferation (Fig. 5C), suggesting that CAR RNAi-mediated activation of p38 MAPK is regulated through an Src-dependent pathway, and Src is involved in CAR RNAi-mediated enhancement of cell migration and proliferation. More intriguingly, *in vivo* RNAi with a ~60% CAR knockdown efficiency (Fig. 6) significantly expedited the wound healing process in rat mid-dorsal skin, while the use of PP2 or SB203580 remarkably prevented this effect (Fig. 7). Notably, H&E staining in Fig. 7C demonstrated that the newly healed wounds result from both re-epithelialization and skin dermal contraction. Green dotted lines indicate basement membrane zone, the region above these lines represents re-epithelialized epidermis, while the region below these lines reflects dermal contraction. On the other hand, overexpression of CAR in HaCaT cells delayed cell migration and proliferation, further confirming results from RNAi study. These findings collectively suggest that loss of CAR accelerates HaCaT cell migration and proliferation, as well as *in vivo* wound healing in rat skin, involving Src-mediated activation of p38 MAPK through an Src-dependent pathway.

Notably, although the half-life of the two involved drugs, namely PP2 and SB203580, was not specified by the manufacturer, drug concentration and treatment duration were carefully selected based on several referential literatures^21^,^22^,^23^ and our initial pilot experiments. As for the stability of siRNA duplexes, CAR protein level at a later time point (60 h *in vitro* or 14 d *in vivo*, respectively) was examined by immunoblotting. Results showed that after RNAi treatment CAR protein level was regained at 60 h (i.e., on day 5.5 post-RNAi transfection began on day 0) *in vitro*, or on day 14 post-wounding *in vivo* as shown in Fig. 2A–C and Fig. 6A–C, respectively. This restoration of CAR expression is important, as long-term lack of CAR may induce tumor progression and detrimental effects. The concentration and treatment duration of siRNA duplexes were also cautiously selected based on our previously published studies^4^,^24^ and pilot experiments in the current study. Although the gene-knockdown effects of CAR RNAi gradually vanished before the end of experiment, its subsequent influences could be last longer.

Src has been indicated in cell proliferation, differentiation, and gene transcription in many epithelia and endothelia^25^. It can be activated at Tyr416 residue via phosphorylation^26^. A recent study revealed that Src activity was inhibited by a wound-induced keratin during keratinocyte migration and tissue repair^27^. Using anti-*p*-Src^[Y416]^, we examined Src activation in HaCaT cells following CAR knockdown and found that CAR knockdown remarkably increased Src activity. Notably, protein phosphorylation within signaling cascades is usually an early event, Src has been shown to be activated within minutes^28^. To completely inhibit Src signaling, we pre-treated HaCaT cells with Src inhibitor PP2 before RNAi, and results showed that PP2 prevented the increase of Src phosphorylation as well as the acceleration in HaCaT cell migration and proliferation, indicating the involvement of Src in CAR knockdown-mediated acceleration of cell migration and proliferation.

Erk1/2, JNK, and p38, the three major groups of MAPKs, are extensively involved in many eukaryote behaviors, like migration and proliferation^29^. A current study showed that the phosphorylation of Erk1/2 and p38 MAPK induced by activation of OR2AT4 were involved in human keratinocyte re-epithelialization during wound healing^30^. The phosphorylation of Erk1/2 together with dephosphorylation of p38 MAPK were also shown to be involved in formononetin-mediated endothelial repair and wound healing^31^. In addition, a study showed that inhibition of Erk pathway totally blocked wound closure and inactivated many early transcription factors, while p38 MAPK inhibition only delayed the healing course in cultured human keratinocytes^32^, which is consistent with our finding that inhibition of p38 by SB203580 significantly slowed down the *in vitro* scratch gap closure. Compelling evidences are accumulating for the close link between the enhanced cell migration and proliferation in cancer^33^, epithelia^34^, and endothelia^35^ with the increased activity of Erk1/2, JNK, and p38 MAPK. In the present study, we have proven that p38 plays a predominant role in the acceleration of HaCaT cell migration and proliferation following CAR knockdown.

Although MAPKs have been implicated in various cellular physiology, little information has been available on how other signaling cascades may influence MAPK activation during wound healing, especially re-epithelialization. Previous studies have linked MAPKs as downstream targets to Src signaling^36^,^37^. In consistence with this, our results have suggested that p38 activation following CAR knockdown in HaCaT cells was dependent on Src activation, while Erk1/2 and JNK were not affected. Additional *in vitro* studies were performed to further elucidate the role of MAPKs, results confirmed that p38 MAPK was critical to HaCaT cell migration and proliferation, but not JNK signaling.

In summary, our study has demonstrated that the activation of Src kinase induced by CAR knockdown results in p38 activation and thus leads to the acceleration in HaCaT cell migration and proliferation, as well as *in vivo* wound healing in rat skin. Nonetheless, further studies are required to delineate the precise mechanisms by which CAR knockdown induces a significant increase in Src kinase activity. Co-IP experiment conducted by Wang *et al.* has revealed the close association between CAR and Src in Sertoli cells^11^. Another study has shown that CAR was associated with microtubules and F-actin, contributed to cytoskeletal equilibrium, and thus inhibited cell migration^38^. Therefore, we believe that the insight into CAR-Src-p38 MAPK signaling is useful for developing pharmacological interventions that are capable to promote wound healing process.

## Materials and Methods

### Ethics statement

The protocol (No: XJYYLL-2013190) for human and animal study was reviewed and approved by the Institutional Ethics Committee of the Fourth Military Medical University. Experiments were carried out in accordance with the approved guidelines. Patients who offered skin samples provided their written, informed consent. Patient samples were deidentified and anonymized prior to various analyses.

### Antibodies

Antibodies were obtained commercially and specifically reacted with target proteins in human and/or rats as indicated by manufacturers (Table S1). The use of antibody for various applications along with the appropriate working dilution was also listed in Table S1.

### HaCaT cell cultures

HaCaT cells were cultured in RPMI 1640 medium (GIBCO, Grand Island, NY) replenished with 10% fetal bovine serum (FBS; GIBCO) and 1% Pen/Step (GIBCO) at 37 °C in an atmosphere of 5% CO_2_. Depending on the type of experiment, HaCaT cells were plated at different cell densities and on different culture dishes for various applications. For immunoblotting analysis, cells were plated at 5 × 10^5 ^cells/cm^2^ on 60-mm dishes with 4-ml culture medium. For immunofluorescence staining, cells were seeded at 5 × 10^5 ^cells/cm^2^ on 24-well dishes with 0.5-ml medium per well. In wound scratch assay, cells were cultured at 1.2 × 10^6 ^cells/cm^2^ in 12-well dishes with 1-ml medium in each well. In MTT assay, cells were plated at 2.5 × 10^5 ^cells/cm^2^ in 96-well dishes with 0.2-ml medium per well. Medium was replaced daily.

### Transfection of HaCaT cells with siRNA duplexes for RNAi experiments

HaCaT cells were cultured for 24 h allowing the full attachment with the inner surface of plates/dishes. Cells reaching ~75% confluence were transfected with 80 nM scramble siRNA duplexes (5′-UUCUCCGAACGUGUCACGUtt-3′, serving as negative control; Thermo Fisher Scientific, Waltham, MA) or CAR-specific siRNA duplexes (#1131: 5′-GAGCAAGGAUGGGUCUAUAtt-3′, #431: 5′-CUCCUGGUGUUGCAAAUAAtt-3′, #106: 5′-GCCAGAAGUUUGAGUAUCAtt-3′, all three sequences react with both human and rat samples; Thermo Fisher Scientific) by using Lipofectamine 2000 Transfection Reagent (Invitrogen, Carlsbad, CA). After 24-h transfection, the reaction mixture was removed, cells were washed and replaced with fresh RPMI 1640 medium containing 10% FBS. Cells were then continuously cultured for another 48 h before termination or use for various applications.

### Treatment of HaCaT cells with Src or p38 inhibitor

HaCaT cells were pretreated with PP2, a putative Src inhibitor (Sigma-Aldrich, St. Louis, MO) at the working concentration of 10 μM, or SB-203580, a p38 inhibitor (Sigma-Aldrich) at the working concentration of 5 μM for 2 h before subjected to *in vitro* RNAi to prevent any Src or p38 signaling. After 24-h transfection, the reaction medium was removed, cells were cultured in fresh RPMI 1640 medium with 10% FBS for another 48 h, and then subjected to subsequent scratch or MTT assay. DMSO was used as vehicle control.

### Western blot analysis

Normal skin tissues (Fig. 1) were obtained from four patients (one male and three females, aged between 20 to 45 years old) during their scar excision surgery in Department of Burns and Cutaneous Surgery, Xijing Hospital. Skin samples were digested with 0.25% dispase II (Roche, Mannheim, Germany) at 4 °C overnight, then the epidermis and dermis were detached. After being washed three times with PBS, approximately 90 mg samples from skin, epidermis or dermis were lysed in RIPA buffer supplemented with protease and phosphatase inhibitor mixtures (Heart Biological Technology Co. Ltd., Xi’ an, China) on ice by using TissueLyser II (Qiagen, Hilden, Germany) for a total 9 min. Tissue homogenates were then centrifuged at 15,000 × *g* at 4 °C for 45 min to obtain total proteins. Lysates from skins surrounding the wounded area on rat back after receiving *in vivo* RNAi treatment (Fig. 6) were collected from adult SD rats (weighting 200~250 g b.w. each) and subjected to western blot analysis to validate if CAR expression was successfully suppressed by RNAi. Lysates from HaCaT cell cultures were directly obtained by using RIPA buffer containing protease and phosphatase inhibitors (Heart Biological Technology), followed by sonication and then centrifuge at 15,000 × *g* at 4 °C for 45 min to obtain clear supernatants. 40 μg lysates from each sample were subjected to SDS-PAGE. Primary antibodies were used as corresponding recommendations (Table S1). Protein estimation was conducted by using a Bradford Protein Assay Kit and a Bio-Rad Model 680 Plate Reader (Bio-Rad Laboratories, Hercules, CA).

### MTT cell proliferation assay

Two days after the completion of RNAi or plasmid transfection, HaCaT cells were cultured for selective time periods: 0, 12, 24, 36, and 48 h. At the end of each time point, cells in each 96-well were added with 20 μl MTT at 5 mg/ml (Millipore, Bedford, MA), and then cultured at 37 °C for 4 h followed by adding 100 μl DMSO to each well. The relative cell number was determined by measuring the O.D. value at 560 nm using a Bio-Rad Model 680 Microplate Reader. MTT assay was done in cells without mitomycin C pretreatment. This experiment was repeated at least four times using different batches of HaCaT cells.

### ***In vitro*** scratch assay

HaCaT cells were cultured for 24 h to achieve 100% confluence followed by starvation in serum-free RPMI 1640 medium containing 10 μg/ml mitomycin C (Invitrogen) for 1 h to completely inhibit cell proliferation. A 200-μl sterile pipette tip was used to make a scratch in HaCaT cell cultures. Cells were then incubated in fresh medium containing 10% FBS for different time periods: 0, 24, 48, and 60 h at 37 °C in a 5% CO_2_ incubator. The scratch gap width at each time point in each treatment group was measured at four different positions and compared to the gap width at 0 h which was arbitrarily set as 1.

### Immunocytofluorescence (ICF) in HaCaT cells

ICF was performed to visually examine the changes on protein localization and distribution in HaCaT cells after RNAi alone or with drug treatment. HaCaT cells were cultured on glass coverslips placed in 24-well dishes. After treatment cells were washed with PBS twice, fixed in 4% paraformaldehyde for 10 min, and permeabilized with 0.1% Triton X-100 for 8 min. After blocking with 1% bovine serum albumin, cells were incubated with target primary antibody at an appropriate dilution at 4 °C overnight. On the next day, cells were incubated with a secondary antibody conjugated with Cy3 (Invitrogen) for 30 min, and then mounted by using Prolong Gold Anti-fade Reagent with DAPI (Invitrogen) for nuclei staining. Immunofluorescence micrographs were obtained by using an Olympus FSX100 Fluorescence Microscope (Olympus, Tokyo, Japan).

### Immunohistochemistry (IHC) and immunohistofluorescence (IHF)

Normal human skin samples were fixed in 4% paraformaldehyde, embedded in paraffin, and subjected to IHC/IHF. Briefly, paraffin sections were cut at 4-μm thickness, rehydrated, blocked, and incubated with anti-CAR primary antibody overnight. On the next day, secondary antibody conjugated with HRP-streptavidin (for IHC) or Cy3 (for IHF) was applied and incubated for 1 h at room temperature. For IHC, DAB was used to visualize CAR immunopositive staining and hematoxylin was used to counterstain nuclei. For IHF, sections were mounted with Prolong Gold Anti-fade Reagent with DAPI for nuclei staining. Images were captured by using the Olympus FSX100 microscope.

### ***In vivo*** wound healing

For wounding experiments, adult SD rats weighting 200~250 g were anesthetized by intraperitoneal administration of pentobarbital (1 g in 100 ml 0.9% NaCl, 5 ml/kg b.w.). Full-thickness wounds at 1.0-cm^2^ (1.0 cm × 1.0 cm) were created on the mid-dorsal skins by using 4-mm disposable biopsy punches on day 0. To evaluate the effect of CAR knockdown on wound healing *in vivo*, 80 nM scramble or CAR-specific siRNA duplexes along with vehicle (DMSO), PP2 (10 μM) or SB203580 (5 μM) were topically injected into skins surrounding the wounds on day 1 and day 3. Images of the wounds were taken on day 1, 4, 7, and 14 post-wounding by a digital camera. The wounded areas were measured by using Image-Pro Plus software. [wound area on day x/wound area on day 1] was defined here as the relative wound area. After sampling, rats were sacrificed by CO_2_ asphyxia.

### HE staining

Day-7 rats post-wounding were selected for HE staining. Full-thickness skins covering the wounds and surrounding areas were excised, fixed in 4% paraformaldehyde for 24 h, embedded in paraffin, and then subjected to HE staining. The unhealed wound width was defined as the distance between two opposite advancing edges of epidermal keratinocyte migration. The entire wounds were artificially reconstituted by overlapping multiple images.

### Overexpression of CAR in HaCaT cells

The full-length cDNA encoding human CAR was obtained by PCR as earlier described^4^ using cDNAs derived from HaCaT cell total RNA (GenBank accession no: Y07593.1) *via* a reverse transcription step that served as the template and a CAR-specific primer pair designated Ex-CAR (Sense: 5′-ATTCCCAGGAGCGAGAGC-3′; Anti-sense: 5′-AGTTCACCCATGTCTTCACCTAT-3′). The full-length CAR cDNA was then cloned into pCI-neo mammalian expression vector (Promega, Madison, WI) at the restriction enzyme sites between *Xho*I and *Not*I by using specific primers of CAR (Sense: 5′-ACCTCGAGATGGCGCTCCTGCTGT-3′; Anti-sense: 5′-ACGCGGCCGCCTATACTATAGACCCA-3′). The pCI-neo mammalian expression vector carries the human cytomegalovirus immediate early enhancer/promoter region that promotes constitutive expression of the CAR insert in HaCaT cells. The authenticity of these clones was confirmed by direct nucleotide sequencing. HaCaT cells were transfected with plasmid DNA by using Effectene Transfection Reagent (Qiagen) at a ratio of 1 μg DNA to 15 μl transfection reagent. Transfection mixture was removed 24 h thereafter and replaced with fresh RPMI 1640 medium with 10% FBS. Protein lysates were extracted from these HaCaT cell cultures 2-day thereafter (i.e., 3-day after transfection began), as described previously.

### Statistical analysis

Statistical calculations were performed with the use of GraphPad Prism software (version 4.0.0.; GraphPad Software, La Jolla, CA). Each experiment was repeated at least four times. Statistical significance was analyzed by one-way ANOVA with Dunnett’s test.

## Additional Information

**How to cite this article**: Su, L. *et al.* Loss of CAR promotes migration and proliferation of HaCaT cells, and accelerates wound healing in rats via Src-p38 MAPK pathway. *Sci. Rep.* **6**, 19735; doi: 10.1038/srep19735 (2016).

## Supplementary Material

Supplementary Dataset 1
