# Supplementary material for: Loss of CAR promotes migration and proliferation of HaCaT cells, and accelerates wound healing in rats via Src-p38 MAPK pathway
Source: Sci Rep. 2016 Jan 25;6:19735. doi: 10.1038/srep19735 (PMC4726158; doi:10.1038/srep19735)
Supplement: Supplementary Dataset 1 [file srep19735-s1.doc]

**Loss of CAR promotes migration and proliferation of HaCaT cells, and accelerates wound healing in rats via Src-p38 MAPK pathway**

Linlin Su^1, #, *^, Lanqing Fu^2, #^, Xiaodong Li^3, #^, Yue Zhang^1, #^, Zhenzhen Li^1^, Xue Wu^1^, Yan Li^1^, Xiaozhi Bai^1^, Dahai Hu^1, *^

^1^Department of Burns and Cutaneous Surgery, Xijing Hospital, the Fourth Military Medical University, Xi’an, Shaanxi 710032, China.

^2^Department of Orthopedics, Jingzhou Central Hospital, Tongji Medical College of Huazhong University of Science and Technology, Jingzhou, Hubei 434020, China.

^3^Department of Burns and Plastic Surgery, General Hospital of Lanzhou Petrochemical Company, Lanzhou, Gansu 730060, China.

^#^These authors contributed equally to the work.

^*^Correspondence should be addressed to:

Linlin Su, linlinsu@fmmu.edu.cn. Department of Burns and Cutaneous Surgery, Xijing Hospital, the Fourth Military Medical University, No.127 Changle West Road, Xi’an, 710032, China. Tel: +86-29-8477 5570, Fax: +86-29-8325 1734.

Dahai Hu, hudhai@fmmu.edu.cn. Department of Burns and Cutaneous Surgery, Xijing Hospital, the Fourth Military Medical University, No.127 Changle West Road, Xi’an, 710032, China. Tel: +86-29-8477 5298, Fax: +86-29-8325 1734.

**Supplementary Materials**


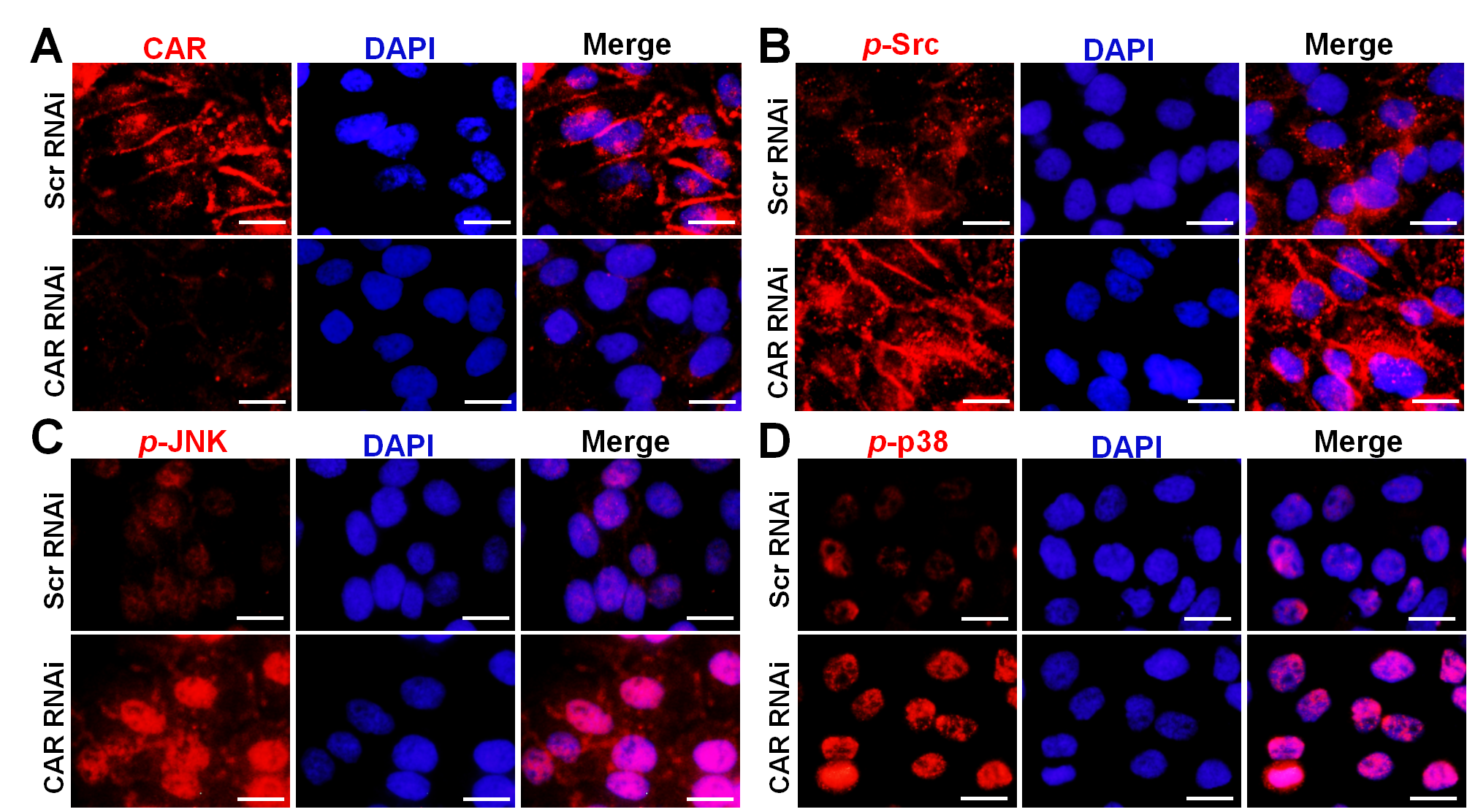


**Figure S1. Study by immunocytofluorescence to assess the effects of CAR knockdown on the expression and localization of selected phosphorylated proteins in HaCaT cells.** HaCaT cells at 5 × 10^5^ cells/cm^2^ were cultured alone for 24 h to allow full attachment and then transfected with scramble or CAR-specific siRNA duplexes for 24 h. Two days thereafter, cells were washed, fixed and processed for immunocytofluorescent staining of CAR (***A***; *red*), *p*-Src (***B***; *red*), *p*-JNK (***C***; *red*) or *p*-p38 (***D***; *red*). Nuclei were visualized with DAPI (*blue*). Scale bar: 20 µm. ‘Scr’, scramble.


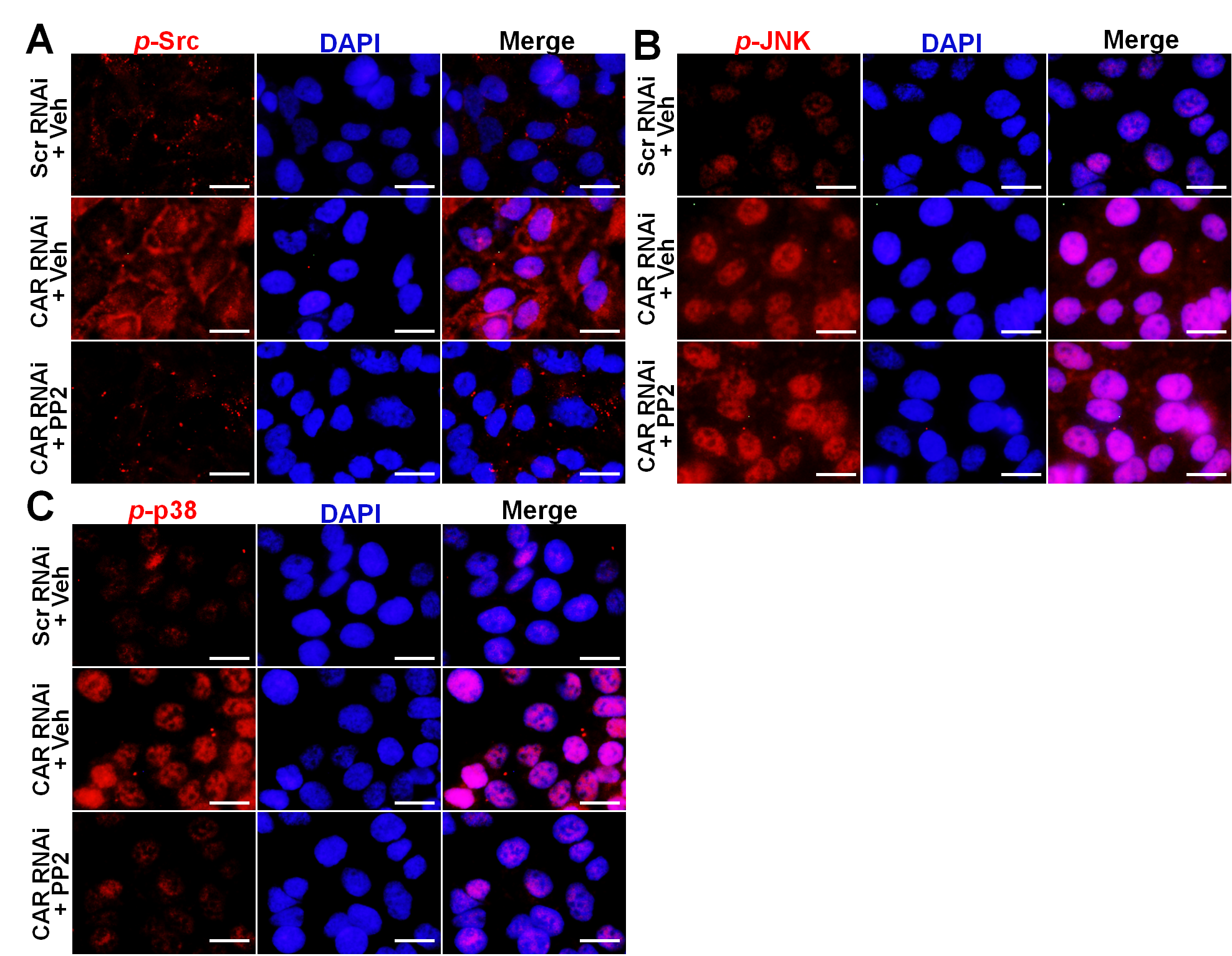


**Figure S2. Study by immunocytofluorescence to assess the effects of PP2 pretreatment on the expression and localization of selected phosphorylated proteins in CAR-silenced HaCaT cells.** HaCaT cells at 5 × 10^5^ cells/cm^2^ were cultured alone for 24 h to allow full attachment and then received vehicle (DMSO) or PP2 pretreatment for 2 h followed by transfection with scramble or CAR-specific siRNA for 24 h. Two days thereafter, cells were washed, fixed and processed for immunocytofluorescent staining of *p*-Src (***A***; *red*), *p*-JNK (***B***; *red*) or *p*-p38 (***C***; *red*). Nuclei were visualized with DAPI (*blue*). Scale bar: 20 µm. ‘Scr’, scramble; ‘Veh’, vehicle.

**Table S1. Antibodies used for different experiments in this study.**

|  |  |  |  |  | **Dilution** | |
| --- | --- | --- | --- | --- | --- | --- |
| **Target protein** | **Catalog #** | **Lot #** | **Host** | **Vendor** | **WB** | **ICF/IHF/IHC** |
| CAR^a^  CAR^b^ | sc-15405  sc-56892 | C1413  / | Rabbit  Mouse | Santa cruz biotechnology  Santa cruz biotechnology | 1:200  1:200 | 1:100/1:50/1:50 |
| c-Src | sc-8056 | F3011 | Mouse | Santa cruz biotechnology | 1:200 |  |
| *p*-Src^[Y416]^ | 6943 | 1 | Rabbit | Cell signaling technology | 1:1000 | 1:100 |
| FAK | sc-558 | E1513 | Rabbit | Santa cruz biotechnology | 1:200 |  |
| *p*-FAK^[Y397]^ | 8556 | / | Rabbit | Cell signaling technology | 1:1000 |  |
| Akt | 4691 | 17 | Rabbit | Cell signaling technology | 1:1000 |  |
| *p*-Akt^[S473]^ | 4060 | 16 | Rabbit | Cell signaling technology | 1:1000 |  |
| Erk1/2 | 4695 | 14 | Rabbit | Cell signaling technology | 1:1000 |  |
| *p*-Erk1/2^[T202/Y204]^ | 4370 | 9 | Rabbit | Cell signaling technology | 1:2000 |  |
| JNK | 9258 | 9 | Rabbit | Cell signaling technology | 1:1000 |  |
| *p*-JNK^[T183/Y185]^ | 4668 | 11 | Rabbit | Cell signaling technology | 1:1000 | 1:100 |
| p38 | 8690 | 1 | Rabbit | Cell signaling technology | 1:1000 |  |
| *p*-p38^[T180/Y182]^ | 4511 | 10 | Rabbit | Cell signaling technology | 1:1000 | 1:100 |
| β-Actin | sc-1616 | B2013 | Goat | Santa cruz biotechnology | 1:200 |  |

Antibodies used herein were known to cross-react with the corresponding proteins in human as indicated by the manufacturers. WB, western blot; ICF, immunocytofluorescence; IHF, immunohistofluorescence; IHC, immunohistochemistry.
